# Diversity and functional traits based indigenous rhizosphere associated phosphate solubilizing bacteria for sustainable production of rice

**DOI:** 10.3389/fmicb.2024.1470019

**Published:** 2024-12-13

**Authors:** Maria Rasul, Mahreen Yahya, Muhammad Suleman, Sughra Hakim, Babur S. Mirza, Muhammad Sajjad Mirza, Thomas Reitz, Mika Tapio Tarkka, Sumera Yasmin

**Affiliations:** ^1^Division of Soil and Environmental Biotechnology, National Institute for Biotechnology and Genetic Engineering College (NIBGE-C), Pakistan Institute of Engineering and Applied Sciences (PIEAS), Faisalabad, Pakistan; ^2^Department of Biological Sciences, Clemson University, Clemson, SC, United States; ^3^Department of Biology, Missouri State University, Springfield, MO, United States; ^4^Department of Soil Ecology, UFZ - Helmholtz-Centre for Environmental Research, Halle, Germany; ^5^German Centre for Integrative Biodiversity Research (iDiv) Halle-Jena-Leipzig, Leipzig, Germany

**Keywords:** Basmati rice, next-generation sequencing, microbial diversity, biofertilizers, nutrient recycling, sustainable agriculture

## Abstract

**Introduction:**

Rice, particularly Basmati rice, holds significant global importance as a staple food. The indiscriminate use of phosphate-based fertilizers during rice production has led to high residual levels of these chemicals in soil, impacting soil health and fertility. This study aimed to address this challenge by investigating the potential of phosphate solubilizing bacteria (PSB) in improving soil fertility and boosting the growth of Basmati rice.

**Methods:**

Using amplicon-based 16S rDNA sequencing, bacterial isolation and cultivation, conducting greenhouse and field experiments, and PSB localization, we optimized the search for PSB inoculants to enhance Basmati rice growth.

**Results and discussion:**

Rice rhizosphere prokaryote communities showed significant differences in microbial diversity and composition between between basmati and non-basmati rice cultivated areas. Dominant bacterial phyla included Proteobacteria, Acidobacteria, Actinobacteria, and Firmicutes, with Actinobacteria and Proteobacteria playing a crucial role in nutrient recycling. Isolation and optimization of PSB strains, including Acinetobacter sp. MR5 and Pseudomonas sp. R7, were carried out and soil microcosm studies confirmed their efficacy in increasing soil available phosphorus concentration. Response surface methodology revealed the relative importance of factors such as pH, inoculum density and incubation temperature in maximising phosphate solubilization. Microplot experiments demonstrated the effectiveness of optimized PSB inoculants in promoting Basmati rice growth, with significant increases in plant height, tiller number, biomass, and grain yield compared to uninoculated controls. A consortium of PSB proved superior to single-strain inoculants, even with reduced chemical fertilizer application. Field trials at several rice growing sites confirmed the positive impact of the PSB consortium on grain yield, soil phosphorus availability, and plant phosphorus uptake. The competence and persistence of the inoculated strains in the rhizosphere was confirmed by FISH and BOX Polymerase Chain Reaction (BOX-PCR). This work highlights the potential of PSB-based biofertilizers to improve soil fertility, promote sustainable rice production and reduce the negative environmental impacts of chemical fertilizers. Future research would focus on scaling up these findings for widespread adoption in agriculture and exploring their applicability to other crops and agroecosystems.

## Introduction

Rice (*Oryza sativa* L.) is the most important food crop in the world and sustains the diets of over 3 billion people, or about half of the global population. Pakistan is one of the world’s top 10 producers of rice. Based on current trends, 4.6 billion people may consume rice by 2025; therefore, the output of grain needs to increase by 20% to keep up with demand (Albahri et al., 2023).

Phosphorus (P) plays an integral role in regulating the metabolism and concomitantly health of plants. Indiscriminate use of phosphate-based chemical fertilizers to meet the plant P requirements in agricultural lands enhanced the residual level of these chemicals in soil and water due to high water solubility and mobility. Aquatic and terrestrial ecosystems’ contamination drastically affects the biogeochemical cycles and different microbiota (microbial population). Excessive and prolonged application of chemical fertilizers has been documented to disrupt the beneficial soil microflora, leading to nutrient deficiency and disease severity due to weakened plant defensive systems. So, one appropriate choice is to use the microbes (Ducousso-Détrez et al., 2024).

The soil dwelling microbial population plays a crucial role in nutrient recycling and, ultimately, enhances soil health and fertility (Jeffries et al., 2018). Plant growth-promoting rhizobacteria (PGPR) may exert beneficial effects by enhancing the bioavailability of nutrients, possibly by nitrogen fixation, phosphate solubilization, etc. (Glick, 1995; Vejan et al., 2016). Phosphate solubilizing bacteria (PSB) solubilize the insoluble phosphate phosphate complexes in the soil and enhance the amount of available P for plant uptake under P deficit conditions (Tripura et al., 2007). Among bacteria, various genera, that is, *Acinetobacter*, *Acromobacter*, *Agrobacterium*, *Aerobacter*, *Bacillus*, *Burkholderia*, *Enterobacter*, *Enterococcus*, *Erwinia*, *Flavobactrerium*, *Pantoea*, *Pseudomonas*, and *Rhizobium* are involved in P solubilization (Kaur and Sudhakara Reddy, 2014). Nowadays, PSB-based biofertilizers are considered a crucial constituent to contribute sustainable production in agro-ecosystems (Mitter et al., 2021; Yahya et al., 2022; Wang et al., 2023; Ducousso-Détrez et al., 2024). The persistence of these PSB is the most important underlying factor of a successful bioinoculant, indicating its ability to compete with indigenous microbes and cope with other abiotic stresses (Finkel et al., 2017). Despite the great potential, the survival or persistence of PSB in biofertilizers is the major bottleneck toward their field scale efficacy under variable environments (Mitter et al., 2021). This is why different commercial biofertilizers did not perform as well in the field as in a greenhouse or a laboratory (Santos et al., 2019; Vassilev et al., 2020).

The population of soil microbes significantly influences the interactions between plants and the soil environment. These interactions may pave the way for the development of new horizons that will help understand the rhizosphere’s complexity and make use of the propagation of beneficial native microbes that aid in nutrient acquisition and promote plant growth (Mahmud et al., 2021). The next-generation sequencing (NGS) based techniques are robust and versatile tools for evaluating the ecology of microorganisms in their native habitat, making it possible to link environmental ecological processes to particular microbe populations (Urszula, 2022). Undestanding soil microbial community can help isolate soil or site-specific microbes for augmentation in the soil. However, unfortunately, the emphasis on deciphering microbial populations from fields and then targeted isolation of required bacterial genera for enhanced nutrient availability of plants is completely missing. To the best of our knowledge, a comprehensive study to delineate rice-associated core microbial communities and microbial drivers under diverse field conditions and geographies is completely lacking, as wheat microbiome studies are either limited to controlled greenhouse conditions (Schlatter et al., 2020) or specific microbial group or a particular plant compartment (Marghoob et al., 2022).

To fill this knowledge gap, we explored the microbiome of the field soil to investigate the microbes that already exist or prevail in native soil and then isolate them, optimize the culture conditions to maximize bacterial efficiency in phosphate solubilization, and augment the soil with these bacteria as biofertilizer. It was thus hypothesized (H1) that soil microbiome data obtained from field soils enable targeted isolations of beneficial PSB for the development of soil-specific biofertilizers. Optimization (H2) of conditions for large-scale, cost-effective inoculum production that will ensure maximum P-solubilization activity of PSB at the field level. Additionally, we anticipated (H3) correlation between improved soil available P and P solubilization by PSB, which consequently would enhance rice growth and grain yield. The findings of this study would be of great significance as they explore the potential of native PSB to enhance phosphorus availability, reduce reliance on chemical fertilizers, and contribute to sustainable agriculture by developing biofertilizers tailored to specific soils, ultimately improving nutrient efficiency and crop yields.

## Materials and methods

### Soil sample collection

**Figure 1:**
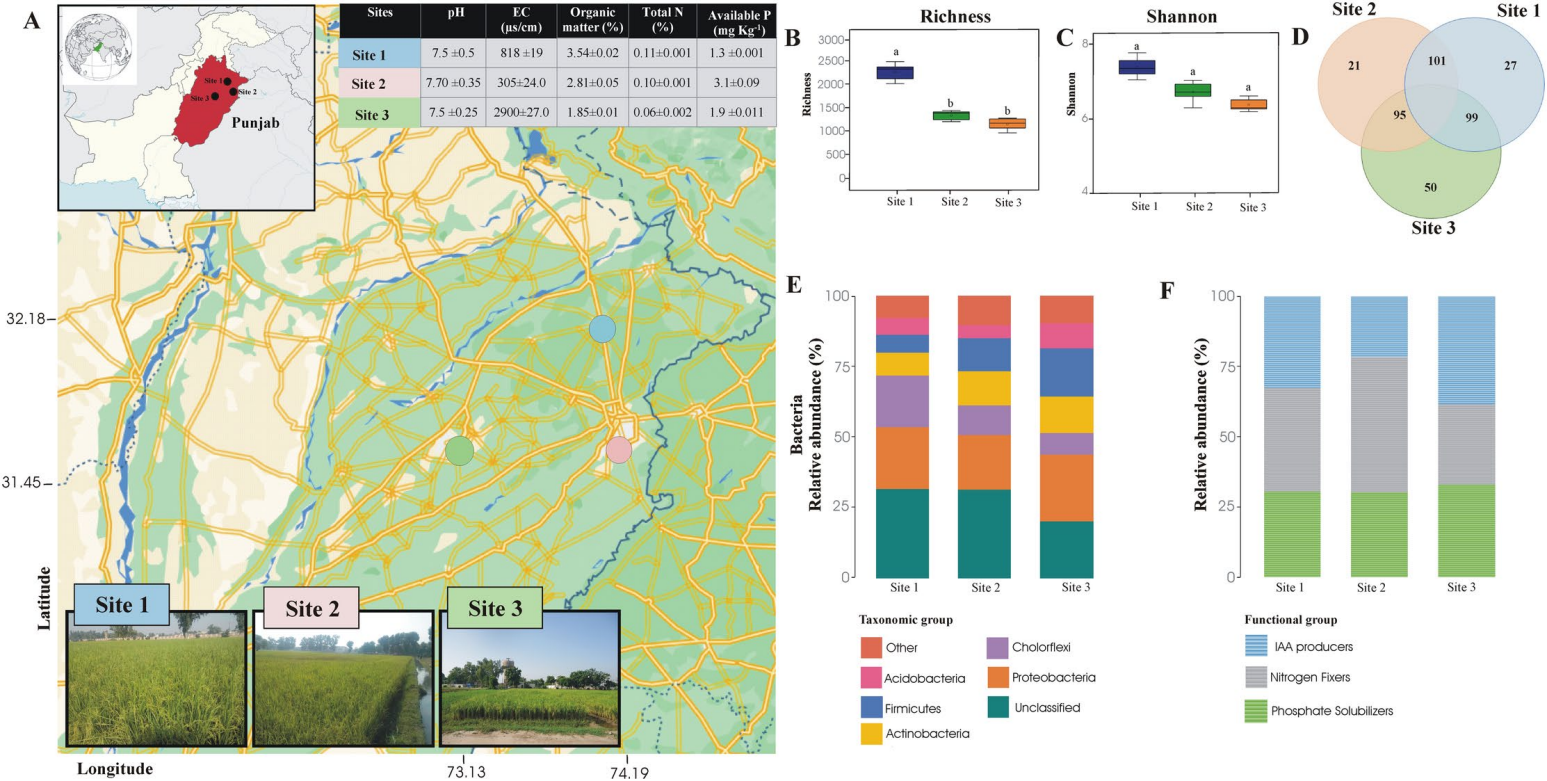
The map shows the positions of the study sites within the chosen region. Each marker indicates where samples were collected for microbiological analysis, covering a range of environmental conditions to ensure thorough data collection **(A)**. Evenness, diversity, and richness of rhizosphere soil sample of Basmati rice **(B)**. Venn diagram showing the bacterial genera shared among the rhizosphere soil samples of Basmati rice collected from different areas of the rice belt and Faisalabad **(C)**. Relative abundance of taxonomic groups **(D)** and functional groups **(E)** detected by Illumina Sequencing of 16S rRNA gene in the rhizosphere soil sample of Basmati rice collected from different areas of rice belt and Faisalabad. Site 1 = Sheikhupura, Site 2 = Gujranwala, and Site 3 = Faisalabad.

Rice rhizosphere soil samples were obtained from experimental locations of adaptive research station Sheikhupura (31°42′42.6”N 73°59′07.5″E), adaptive research station Gujranwala (32°13′21.5”N 74°13′02.7″E) and NIBGE field, Faisalabad (31°23′44.0”N 73°01′37.3″E), for bacterial community analysis and isolation of PSB. Site 1 (Sheikhupura) and site 2 (Gujranwala) are two significant areas of the Basmati rice growing belt, while site 3 (Faisalabad) is out of the traditional rice growing belt (Figure 1A). Ten plants were uprooted from various fields at each site for rhizosphere soil sampling. The loosely attached soil was removed from all plants, and the soil adhering to the roots was collected and combined to create a composite sample. The physicochemical properties of a sub-sample of soil (0.5 kg) were determined (Supplementary Table S1).

### DNA extraction, amplicon library preparation, and sequencing

For genomic DNA extraction from rhizosphere soil, 0.5-g air-dried soil samples were processed using a mechanical lysis, and a DNA isolation kit (MP Biomedicals, Santa Ana, CA, USA) was used according to the manufacturer’s instructions. The variable region (V3–V4) of the 16S ribosomal RNA (rRNA) gene was amplified and sequenced using Illumina MiSeq pair-end DNA sequencing at Utah State University Center for Integrated Biosystems (CIB), Logan, USA.

### Bioinformatics analysis

The data were statistically analyzed using ANOVA, and differences across treatments were assessed with the least significant difference (LSD) test at a 5% confidence level, using STATISTIX 10.0 software (Tallahassee, FL, USA). The phyloseq software^1^ imported the corresponding bacterial OUT matrices, taxonomy tables, and representative sequences into R version 4.0.2 (McMurdie and Holmes, 2013). Coordinate analysis (CA) was performed to investigate the relationship between bacterial phyla at three sites through R software (R version 4.0.2)^2^. 16S rRNA gene sequences were submitted as Fastq files to the NCBI Sequence Read Archive (SRA) with the BioProject ID PRJNA574892.^3^

### Isolation of phosphate solubilizing bacteria

Potential PSB strains were isolated from all three sites of the province Punjab, Pakistan rice growing area based on metagenomic results of dominant genera on selective media for *Bacillus*, *Acinetobacter*, and *Pseudomonas*.

The two most efficient bacteria *Acinetobacter* sp. MR5 (Gen Bank accession number LT629134) and *Pseudomonas* sp. MR7 (GenBank accession number LT629136) was isolated, sequenced based on 16S rRNA sequencing, and screened P solubilizing activity from rice growing area of province Punjab, Pakistan (Rasul et al., 2019). Before applying the bacteria in field trials, *in vitro* studies were conducted to verify their efficiency under various physical parameters under submerged and natural soil conditions.

### Microcosm studies of PSB for P solubilization efficiency in sterilized soil

The experiment aimed to investigate the ability of inoculated *Acinetobacter* sp. MR5 and *Pseudomonas* sp. MR7 to survive and solubilize insoluble phosphate in soil (under controlled circumstances). Tricalcium phosphate (TCP; 0.5 g 50 g^−1^ soil) and glucose (1 g 50 g^−1^ soil) were added to 50-g soil (loamy, pH 7.9, electrical conductivity (EC) 2.1 mS, organic matter 0.85%, soil total P 40 mg kg^−1^, available P 1 mg kg^−1^, total soil N 0.07%, available NH_4_ 0.04%, NO_3_ 0.01%) in 200-ml Pyrex glass bottles (Pyrex, manufactured by Corning Inc., Corning, NY, USA) of microcosm. The 5 mL (109 CFU ml^−1^) culture of each cultivated bacterium was harvested at 28 ± 2°C and 100 rpm in a nutrient broth medium to create the bacterial inocula. The treatments applied were as follows: (1) soil inoculated with *Acinetobacter* sp. MR5, (2) soil supplemented with TCP and inoculated with *Acinetobacter* sp. MR5, (3) soil supplemented with TCP, glucose, and inoculated with *Acinetobacter* sp. MR5, (4) soil inoculated with *Pseudomonas* sp. MR7, (5) soil supplemented with TCP and inoculated with *Pseudomonas* sp. MR7, (6) soil supplemented with TCP, glucose, and inoculated with *Pseudomonas* sp. MR7, (7) soil without inoculation, (8) soil supplemented with TCP (control), and (9) soil supplemented with TCP and glucose (control). For every treatment, three duplicates were set up in a fully randomized design (totaling 81 microcosms). For 60 days, the microcosms were cultured at 28 ± 2°C. Throughout the experiment, the weight of a microcosmic unit was measured every 5 days to ensure that the soil’s moisture content remained at 70% water-holding capacity using sterilized water (up to 60 days). Data were collected on the 15th, 30th, and 60th days following vaccination (DPI). To create a homogenized matrix for additional analysis, obtained soil samples were thoroughly mixed at each step. Using the plate dilution method, the bacterial population was determined from 1 g of soil from each treatment (Somasegaran and Hoben, 2012). The bicarbonate approach was used to calculate the available P in the soil (Olsen, 1954). Using p-nitrophenyl as a substrate to produce a light pink color, phosphatase activity was examined and measured using a spectrophotometer (Malcolm, 1983; Zhu et al., 2017).

### Optimization of the cultural conditions

The significant impact of bacteria on P release in a soil microcosm system facilitated the examination of environmental stressors affecting bacterial efficiency in phosphate solubilization for practical field applications. The study investigated how temperature, initial pH, and culture volume influence P solubilization by specific PSB strains, *Acinetobacter* sp. MR5 and *Pseudomonas* sp. MR7, using Response Surface Methodology (RMS). This was conducted through 23 full factorial Central Composite Designs (CCD) with 20 runs, where variables (a) pH, (b) temperature, and (c) inoculum density (Supplementary Table S2) were manipulated and analyzed. A Central Composite Design with three variable factors and six central points validated the model predictions for P solubilization. The response, P solubilization, was assessed after 3 days of incubation with the two phosphate solubilizing strains.

For experimentation, bacterial strains *Acinetobacter* sp. MR5 and *Pseudomonas* sp. MR7 was grown in nutrient broth for 24 h. Overnight-grown cultures were harvested and centrifuged for 10 min at 8,000 rpm. The supernatant was discarded, and cell pallets were washed 2 times with normal saline solution (0.9% NaCl), re-dissolved in the saline solution, and diluted to adjust the optical density for inoculation.

Various inoculum concentrations recommended by the model were added to 50 mL of Pikovskaya broth medium in triplicate to quantify P. The medium’s initial pH (ranging from 5 to 9) was adjusted according to the model’s specifications. Pikovskaya’s broth cultures containing PSB strains were incubated at temperatures ranging from 25 ± 2°C to 50 ± 2°C on a shaker (Kottermann 4,020, Arnsberg, Germany) at 90 rpm. After 3 days of incubation, the cultures were centrifuged at 4,000 rpm for 10 min at 4°C, and the pH of the supernatant was measured. The quantitative estimation of P released from inorganic phosphates was performed using the vando molybdate method (Murphy and Riley, 1962). The soluble phosphate was measured using a KH_2_PO_4_ standard curve. To determine the amount of solubilized phosphate, a graph of concentration (μg ml^−1^) was plotted against the standard solution (Soltanpour and Workman, 1979).

### Kinetics of phosphate solubilization by PSB

Additional research was done on *Acinetobacter* sp. MR5 and *Pseudomonas* sp. MR7 to determine the best growth kinetic parameters. Furthermore, the effect of inoculum density on growth medium, pH, and P kinetics of P solubilization over a 36-h period was investigated. An equivalent volume of inoculum (10^8^ CFU ml^−1^) cultured in Luria Bertani (LB) broth: Procured from (specific manufacturer, e.g., Sigma-Aldrich, USA) for 36 h was added to the growth medium. To calculate the wet cell weight or packed cell mass of the inoculum, duplicate samples were extracted aseptically, centrifuged for 5 min, and the wet cell mass was subtracted from the weight of the empty eppendorf tube (Eppendorf SE, Hamburg, Germany). Inoculum densities for *Acinetobacter* sp. MR5 were 0.022 and 0.038% (w/v), and 0.008 and 0.014% (w/v).

Time course aliquots in triplicate were aseptically removed at various time intervals (4, 10, 24, and 36 h), and three flasks of each treatment were utilized to measure the degree of phosphate solubilization in relation to cell mass. The influence of pH on the growing media was also investigated. The phosphorus solubility under submerged conditions was evaluated on the same day as stated in the preceding experiment (TCP solubilization in liquid media). Aiba et al. (1973) and Pirt (1975) presented numerous growth kinetics characteristics. The slope of the plot of ln(*x*) vs. time (*t*) was used to calculate the specific growth rate (*μ*), also known as the rate of growth per unit cell mass.

### Evaluation of PSB for rice yield parameters

#### Microplot experiment

*Acinetobacter* sp. MR5 and *Pseudomonas* sp. MR7 was further evaluated in microplots for plant inoculation studies. These PSB were tested on the rice variety, Super Basmati, at the National Institute for Biotechnology and Genetic Engineering (NIBGE) under net house condition in 2015–2016. A total of 2.25 m^2^, six replicate microplots were used for each treatment. The experiment was arranged in a randomized complete block design with five treatments, including two strains of inoculum separately, their consortia, and uninoculated controls with 100% Diammonium Phosphate (DAP) recommended dose, 80% DAP recommended dose. Approximately 80% of DAP dose was used in inoculation treatments. The recommended dose of sterilized filter mud 1 Kg per 50 Kg of seeds was used as carrier material. PSB strains were inoculated along with carrier material at transplantation by root dip method (Tariq et al., 2007; Yasmin et al., 2017). N as urea and P as DAP were applied at 140 and approximately 60 kg acre^−1^ (full and 80% of the recommended dose, respectively). At harvest, data for various plant yield parameters were recorded.

#### Multilocational field trials

A consortium of PSB MR5 and MR7 was further evaluated for its effect on plant growth and P-uptake at three rice cultivation sites: Site 1 in Sheikhupura, Site 2 in Gujranwala, and Site 3 in Faisalabad. Root inoculation of rice seedlings with PSB was performed as described previously. Inoculated and un-inoculated treatments were transplanted into designated plots. The sizes of plots were 3.5 m × 6.5 m at site 1, 5 m × 6.5 m at site 2, and 4 m × 6 m at site 3, with spacing of 23 cm between rows. Flooded conditions were maintained in plots. Treatments contained both recommended levels of N and P (100:70 kg NP ha^−1^), which served as positive controls and controls with no PSB inoculation but 100 and 80% of the recommended NP fertilizer doses. These treatments were distributed in three repetitions using a randomized complete block design (RCBD). DAP was employed in two doses, while urea was applied in three doses: first, before planting during plot preparation, then 25 days after transplantation, and finally 50 days after transplantation.

At harvest, rice plants from each plot were carefully uprooted and cleaned to remove soil. The number of tillers per plant and plant height were recorded. Shoots and roots are separated and weighed after oven drying for 72 h at 70°C. Both grain and straw weights were measured after threshing the entire plant.

#### Detection of inoculated PSB from rice rhizosphere

Several techniques, such as viable cell count (Somasegaran and Hoben, 2012), fluorescent *in situ* hybridization (FISH) with confocal laser-scanning microscopy (CLSM) (Amann et al., 1995), BOX-PCR fingerprinting (Basheer et al., 2016), and plant growth promoting attributes (Suleman et al., 2018; Rasul et al., 2019) were employed to determine the survival of the inoculated PSB.

### Data analysis

Using STATISTIX 10.0 software (Tallahassee, FL, USA), the data were statistically analyzed using ANOVA and the least significant difference (LSD) test at a 5% confidence level to examine the differences between the treatments. Using Design Expert software (State Ease, USA), regression and response surface analysis were used to assess the impact of several parameters (pH, temperature, and inoculum density) on phosphorus solubilization. Principal component analysis (PCA) and categorical-principal component analysis (CAT-PCA) were performed using SPSS software package version 23.0 (SPSS, Inc., Chicago, IL, USA).

## Results

### Bacterial diversity in rhizosphere of Basmati rice

Bacterial diversity from the rhizosphere of two rice-growing sites (sites 1 and 2) was investigated and compared with the bacterial community of a non-Basmati rice-growing site (site 3, Faisalabad) using 16S rRNA gene sequence analysis. A total of 20,069 sequences of the 16S rRNA gene were retrieved, of these, 6,485 sequences were from site 3 (Faisalabad), 5,174 from site 2 (Gujranwala), and 8,410 from site 1 (Sheikhupura). The Operational Taxonomic Unit (OTU) matrices were rarefied to reduce the noise, and the spurious taxa were removed from the 3% of the sample in whole bacterial datasets (Supplementary Figure S1). The bacterial richness and Shannon diversity index for site 1 (Sheikhupura) were significantly higher than for sites 2 and 3. Evenness in the rhizosphere soil samples from sites 1 and 2 was also higher than in site 3 (Supplementary Table S3).

The dominant taxonomic group retrieved from all three sites were Proteobacteria, Acidobacteria, Actinobacteria, Chloroflexi, and Firmicutes. The dominant species at all three sites was Proteobacteria, encompassing 24% of the total sequences from site 3, 22% from site 1, and 19% from site 2. Following Proteobacteria, Firmicutes (17%), Actinobacteria (12%), and Chloroflexi (18%) were dominant species in sites 1–3, respectively. Chloroflexi was found to be less abundant phyla at sites 1 and 2 (10 and 7.6%, respectively) than at site 3 (18%). In contrast, Actinobacteria, Acidobacteria, and Firmicutes were more abundant at sites 2 and 3 than at site 1 (Figure 1).

### Relative distribution of bacteria in rhizosphere at genus level

A total of 159 genera were identified in the rhizosphere soil of site 3, 131 in site 2, and 146 in site 1. At the genus level, the highest number of shared genera (101) was found between sites 1 and 2. Additionally, 50 genera were unique to site 3, while 27 and 21 genera were unique to sites 1 and 2, respectively (Figure 1D).

### Relative abundance of plant growth promoting genera

The total number of sequences retrieved from three different soils (Figure 1F) showed that among the 227 genera identified, 15 were recognized as potential phosphate solubilizers, nitrogen fixers, and Indole-3-Acetic Acid (IAA) producers. Among P solubilizing bacteria, *Bacillus* was the dominant genus in the rhizosphere soil from Site 3, followed by *Promicromonospora* and *Agromyces*. In site 2, *Bacillus* was found to be the dominant genera, followed by *Paenibacillus* and *Mycobacterium*. However, in site 1, both *Bacillus* and *Thiobacillus* were the predominant genera (Figure 1E).

For nitrogen-fixing bacteria, the dominant genera in soil from site 3 were *Promicromonospora, Ramlibacter*, *Paenibacillus*, and *Ensifer*. In soils from sites 2 and 1, the dominant nitrogen-fixing genera were *Nitrospira*, *Clostridium*, and *Bradyrhizobium*.

Potential IAA-producing genera identified in the soil from site 3 included *Agromyces*, *Bacillus*, *Paenibacillus*, *Promicromonospora*, *Pseudonocardia*, and *Mesorhizobium*. In site 2, the dominant IAA-producing genera were *Bacillus*, *Paenibacillus, Pseudonocardia*, and *Streptomyces*. In site 1, the dominant genera were *Bacillus, Agromyces, Aeromicrobium,* and *Paenibacillus* (Figure 1E). Seven common bacterial genera with potential for IAA production, P solubilization, and nitrogen fixation were identified: *Azospirillum*, *Bacillus*, *Brevibacillus*, *Mesorhizobiuiaam*, *Paenibacillus*, *Streptomyces*, and *Sphingomonas*.

### Bacterial strains

Bacterial strains *Acinetobacter* sp. strain MR5 (GenBank accession number LT629134; DSMZ accession number 106631) and *Pseudomonas* sp. strain MR7 (GenBank accession number LT629136; DSMZ accession number 106634), were isolated from rice-growing areas in Punjab, Pakistan, and selected based on their phosphate-solubilizing potential (Rasul et al., 2019). Before applying these bacterial strains in field trials, an *in vitro* studies were conducted to ensure their efficacy in submerged and natural soil conditions under variable physical conditions.

### Microcosm studies for evaluation of PSB in sterilized soil

**Figure 2:**
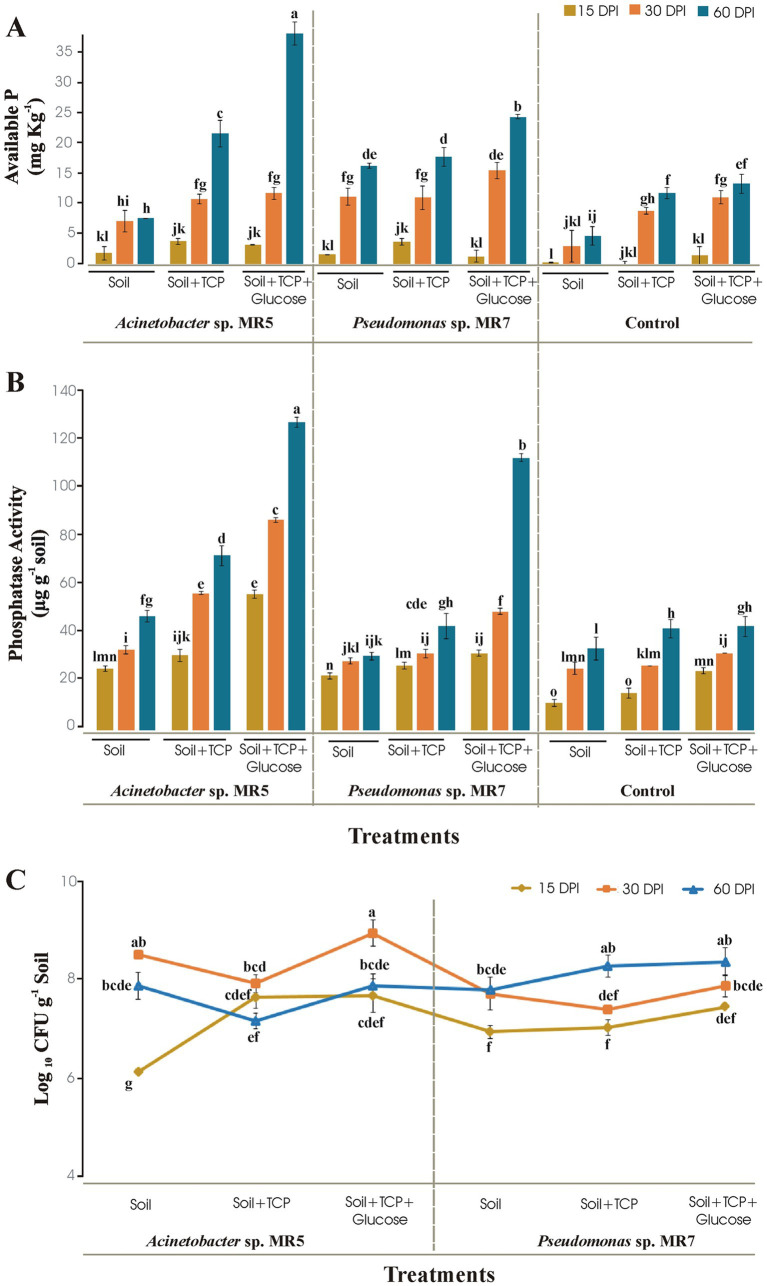
Soil microcosm to study the effect of *Acinetobacter* sp. MR5 and *Pseudomonas* sp. MR7 at 15 DPI, 30 DPI, and 60 DPI on **(A)** soil available P **(B)** bacterial phosphatase activity and **(C)** total bacterial population. Means are an average of three biological replicates and means followed by the same letter differ non-significantly at *p* = 0.01 according to LSD using 3-way ANOVA.

*Acinetobacter* sp. MR5 and *Pseudomonas* sp. MR7 was investigated for P solubilization in sterilized soil at different time intervals under controlled conditions. *Acinetobacter* sp. MR5 solubilized the maximum available phosphorus (34.45 mg kg^−1^) while *Pseudomonas* sp. MR7 showed 22 mg kg^−1^ available P in soil enriched with TCP and glucose at 60 DPI. Available phosphorus was 12 mg kg^−1^ at 60 DPI in their respective un-inoculated control treatment (Figure 2A). Phosphatase activity showed by *Acinetobacter* sp. MR5 and *Pseudomonas* sp. MR7 was124 μg g^−1^ and 110 μg g^−1^, respectively, in soil enriched with TCP and glucose at 60 DPI (Figure 2B). An increase in the population of beneficial bacteria in soil showed bacterial competency, and enhanced soil fertility by increasing essential nutrient availability (N, P, K, etc.). Bacterial count of *Acinetobacter* sp. MR5 inoculation was found to be maximum (8.86 log_10_ CFU g^−1^) after 30 Days Post Inoculation (DPI) in soil supplemented with TCP and glucose, while the bacterial count of *Pseudomonas* sp. MR7 inoculation was found to be maximum (8.08 log_10_ CFU g^−1^) after 60 DPI in the soil was supplemented with TCP and glucose (Figure 2C). Un-inoculated control treatments showed no bacterial growth.

### Optimization of culture conditions using RSM

Response Surface Methodology was used with 20 experimental runs to evaluate the interaction effects of pH, temperature, and inoculum density on phosphate solubilization, in contrast to the conventional “one factor at a time” approach. Eight factorial points, six axial points, and six central point’s made up the experimental configuration based on a CCD. The following equations demonstrated the phosphate solubilization activity of both bacterial strains:


$$\begin{array}{l} MR5.Y=+4.51-0.22A-0.94B\\ +0.16C-0.21\mathrm{AB}+0.022\mathrm{AC}\\ -0.072BC-0.36A2-0.19B2-0.33C2 \end{array}$$

$$\begin{array}{l} MR7.Y=+119.29-5.09A-16.94B\\ +11.6C-18.63\mathrm{AB}+0.13\mathrm{AC}\\ -0.88BC-24.64A2-9.79B2-11.38C2 \end{array}$$

Where the factors A, B, and C are coded, and *Y* denotes the expected response. According to multiple linear regression, the investigated factors had linear, intrinsic, and quadratic impacts. Each variable’s synergistic and antagonistic effects are indicated by the positive and negative signs, respectively.

The “Lack of Fit *F*-value” (0.60) with a *p*-value of 0.71 suggested a non-significant lack of fit for *Acinetobacter* sp. MR5, but the Model *F*-value (80.14) indicated significance. The ANOVA findings validated the model’s significance, which also supported the model fit with adjusted *R*^2^ (0.99) and projected *R*^2^ (0.97). The three variables (temperature, pH, and inoculum size) had very significant interactive effects on P solubilization activity, with all three variables having *p*-values less than 0.01. Bacterial activity may be decreased by deviations from the optimal conditions (Supplementary Tables S4, S5).

Regarding *Pseudomonas* sp. MR7, the quadratic source model displayed an insignificant lack of fit (*F*-value: 3.91), whereas the Model *F*-value (373.7) indicated relevance. The *R*^2^ value (0.99) and expected *R*^2^ (0.98) further confirmed the model fit. Key terms in the model were A, B, C, AB, A^2^, B^2^, and C^2^. These were limiting constraints, and even little changes in these parameters could impact the solubility of phosphate by the bacteria.

### Response surface plots for solubilization of phosphate

**Figure 3:**
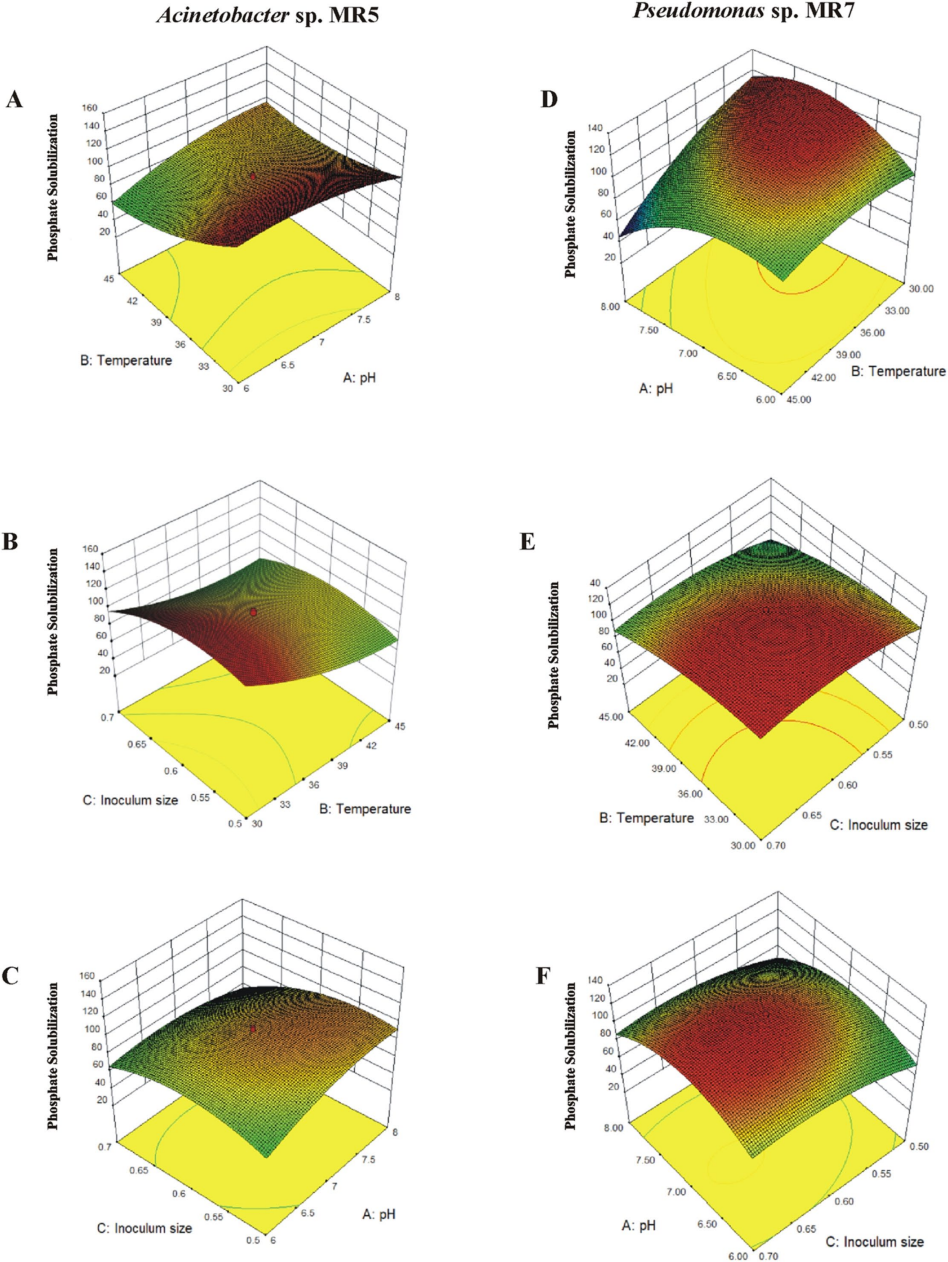
The interaction of different variables in response to inoculation of *Acinetobacter* sp. MR5 and *Pseudomonas* sp. MR7 on solubilized phosphate. 3D surface curves were plotted against two independent variables and keeping other variable at their central (0) level. The 3D curves and contour plots from the interactions between variables of the calculated response are **(A,D)**: relative effect of pH and temperature on phosphate solubilization while keeping inoculum concentration at its central level. **(B,E)** relative effect of temperature and inoculum size on phosphate solubilization while keeping temperature at its central level. **(C,F)** relative effect of pH and inoculum size on phosphate solubilization while keeping pH at its central level.

The 3D surface graphs illustrated the interactive effects of different variables for *Acinetobacter* sp. MR5. Phosphate solubilization increased up to a point when the inoculum size was maintained at an intermediate level while temperature (30–45°C) and pH (6–8) changed. Below that point, a drop was seen. The circular contour plots between pH and temperature demonstrated a substantial interaction impact on phosphate solubilization. The effects of inoculum size and temperature were statistically significant when pH was maintained at an intermediate level with varying inoculum size (0.5–0.7) and temperature. Furthermore, there were notable interactions between pH and inoculum size. At pH 7, phosphate solubilization reached 160 μg L^−1^ with 0.6 g L^−1^ inoculum, increasing with inoculum size (up to 0.68) and optimal temperature (Figure 3).

In *Pseudomonas* sp. MR7, phosphate solubilization increased (up to 120 μgL^−1^) to an ideal point, declining beyond 37.2°C, when the inoculum size was retained at an intermediate level and temperature and pH changed. Significant interaction effects were shown by the contour plots between pH and temperature, which were elliptical. There were notable interactions between inoculum size and pH (6.5–7.5) and substantial effects of temperature and inoculum density. At pH 7, 118 μgL^−1^ of phosphate solubilization was achieved using an inoculum of 0.6 gL^−1^ at 25–37.5°C.

Experiments employing the optimized predicted values from RSM were used to confirm the validity of the applied model. Maximum solubilization was achieved near the anticipated values, indicating the model’s suitability for the particular activity (Figure 3).

### Growth kinetics of phosphate solubilizing bacteria

**Figure 4:**
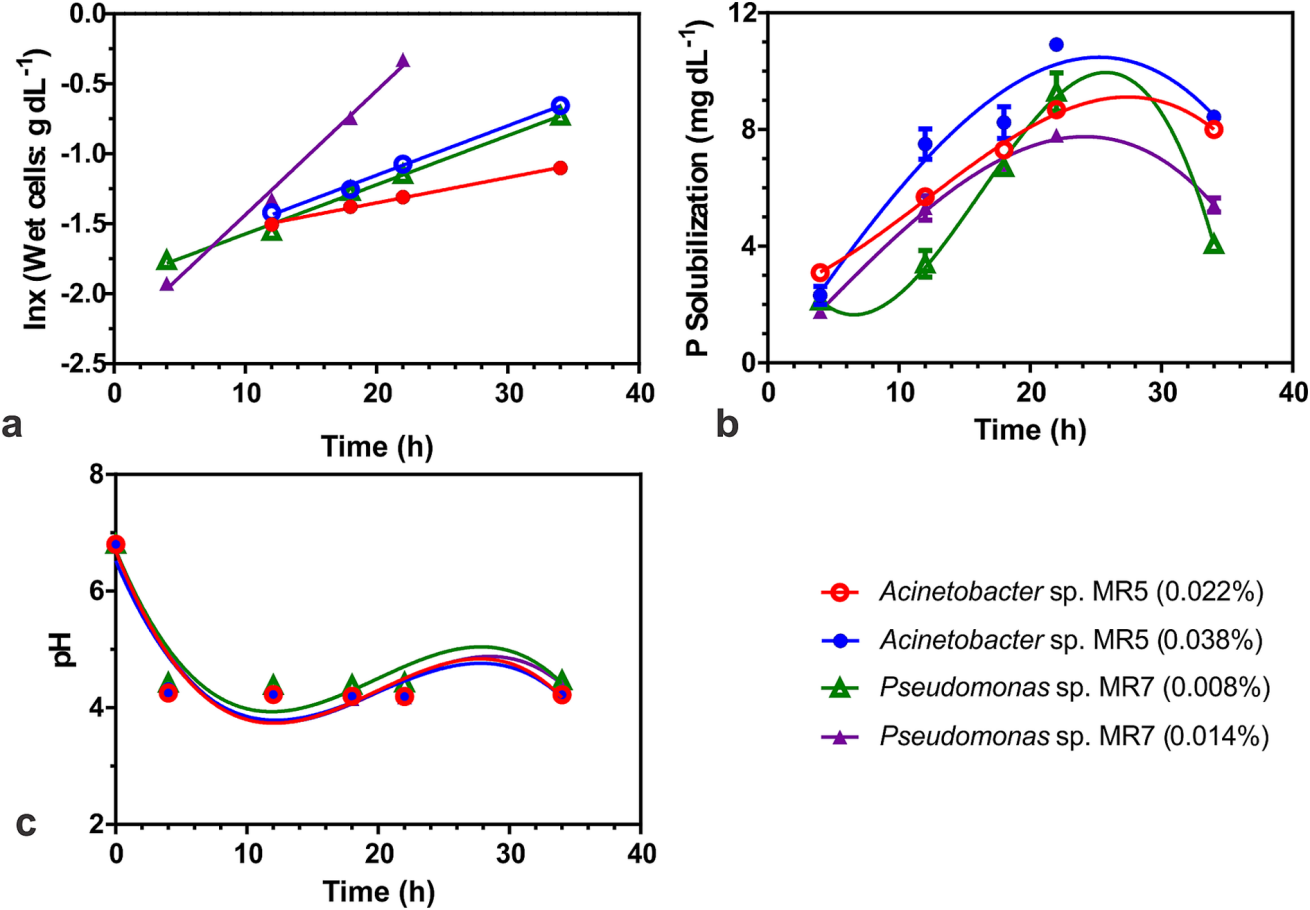
Biokinetics to study the phosphate solubilization activity of two *Acinetobacter* sp. MR5 and *Pseudomonas* sp. MR7. Effect of inoculum on **(A)** Specific growth rate. **(B)** P solubilization **(C)** effect of inoculum on pH. Acinetobacter sp. MR5 (0.022 and 0.038% inoculum w/v wet cells) and *Pseudomonas* sp. MR7 (0.008 and 0.014% inoculum w/v wet cells) were used for inoculation in Pikovskaya broth and kept at 36 ± 2°C. The supernatant was collected at different time intervals (4, 12, 18, 22, and 34 h) and plotted against the time for activity. Means are an average of three biological replicates, ± represents standard deviation.

When grown under submerged conditions, *Acinetobacter* sp. MR5 and *Pseudomonas* sp. MR7 exhibited reduced doubling times (*t*_d_) with increased inoculum density. *Pseudomonas* sp. MR7 had a lower generation time (7.8 h) compared to *Acinetobacter* sp. MR5 (19.6 h). *Acinetobacter* sp. MR5 showed higher phosphorus solubilization (10.86 mg dL^−1^) and a higher specific rate of phosphorus solubilization (qp; 1.12 mg g^−1^ cells h^−1^) than *Pseudomonas* sp. MR7 (Figure 4).

### Evaluation of PSB for P uptake and rice yield

#### Microplot experiment

In a microplot experiment, rice yield parameters were significantly improved by inoculating rice plants with *Acinetobacter* sp. MR5, *Pseudomonas* sp. MR7, and their consortia under net house conditions at NIBGE, Faisalabad. In comparison to both 80 and 100% uninoculated controls, the inoculated plants showed higher plant height (134–136 cm), tillers (18.8–19.7), biomass (3.64–3.82 kg/plot), straw yield (2.84–3.0 kg/plot), and grain yield (0.38–0.39 kg/plot). Furthermore, compared to controls (added with 80 and 100% DAP), the phosphorus (P) content of plants increased in those inoculated with MR5 (0.198%), MR7 (0.20%), and the consortium (0.20%) (Supplementary Figure S2).

#### Multilocational field trials

Inoculating the rice with a consortium comprising MR5 and MR7 improved various plant growth parameters. An increase (11%) in grain yield was observed at Faisalabad, followed by 9% at site 1 and 6% at site 2, compared to the 80% uninoculated control. The highest straw yield increase was 13% at site 1 and 10% at site 3. Overall, the consortium application led to a 4–7% increase in both grain and straw yield compared to the 100% uninoculated control plots. Site 2 recorded the highest grain yield (5,450 kg ha^−1^) and straw weight (16,795 kg ha^−1^) with 20% reduced fertilizer application (Table 1).

**Table 1:** Effect of PSB consortia on various yield and soil parameters in multilocation field trials.

Sites	Treatments	Number of tillers (tillers m^−2^)	Plant height (cm)	Grain weight (Kg ha^−1^)	Plant seed P (%)	Soil available P
Site 1	80% Control	24 ± 1.1 B	114 ± 3.0 B	4828 ± 202 B	3.30 ± 0.20 B	5.23 ± 0.20 B
	Inoculated	25 ± 1.3 A	118 ± 3.5 A	5259 ± 215 A	4.40 ± 0.32 A	6.07 ± 0.34 A
	100% Control	24 ± 1.1 B	118 ± 3.3 A	4928 ± 300 B	4.19 ± 0.23 A	5.51 ± 0.29 B
Site 2	80% Control	25 ± 1.5 B	115 ± 3.0 A	4611 ± 245 B	3.49 ± 0.17 C	5.21 ± 0.25 B
	Inoculated	30 ± 2.0 A	120 ± 4.8 A	5430 ± 300 A	4.70 ± 0.20 A	6.72 ± 0.33 A
	100% Control	29 ± 2.0 A	118 ± 3.5 A	5210 ± 200 A	4.09 ± 0.23 B	5.51 ± 0.28 B
Site 3	80% Control	17 ± 1.5 A	95 ± 3.2 C	3650 ± 210 B	2.80 ± 0.14 B	6.10 ± 0.31 C
	Inoculated	20 ± 1.0 A	99 ± 4.1 A	4299 ± 270 A	4.00 ± 0.26 A	6.69 ± 0.38 A
	100% Control	19 ± 1.0 A	97 ± 3.4 B	3800 ± 224 AB	3.90 ± 0.18 A	6.27 ± 0.27 B

Data is an average of three replicates. ^1^Plant P content is given in % of total plant weight. ^2^Soil available P is presented in μg g−1 soil. Means with significant differences (*p* < 0.05) among treatments are represented by different letters. Site 1 = Sheikhupura, Site 2 = Gujranwala, and Site 3 = Faisalabad.

**Figure 5:**
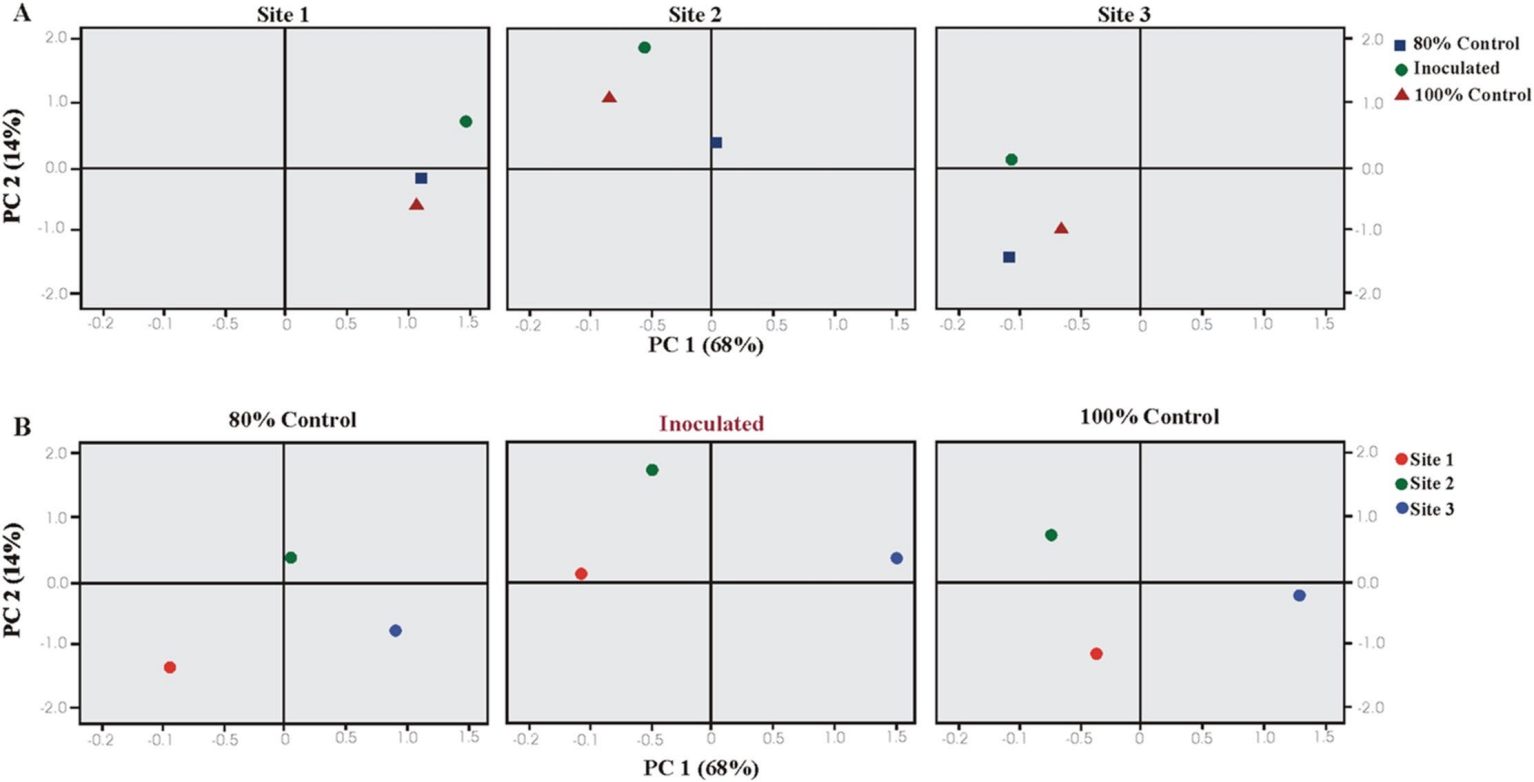
Principal component analysis (PCA) showing the response of bacterial inoculation treatments at three different soils: **(A)** Loaded as soil type (upper panel) and **(B)** treatments (lower panel) (total variance explained: 82%). T1 = 80% uninoculated control, T2 = 80% PSB inoculated treatments, and T3 = 100% uninoculated control. S1 = Sheikhupura, S2 = Gujranwala, and S3 = Faisalabad.

Plant growth parameters were analyzed using PCA and CAT-PCA. The first two components, PC1 and PC2, captured over 82–94% of the total variance. The analysis highlighted significant treatment effects at Sites 1 and 2, with a pronounced effect of PSB inoculation at Sheikhupura soil (S2). PCA indicated that soil and inoculated treatments significantly influenced the majority of the rice growth parameters, showing strong correlations and positive loading on PC1 (Figure 5).

#### Effect of bacterial consortium on P contents of rice

The application of the bacterial consortium significantly increased plant P content (Table 1), with a progressive increase as P levels increased from 80 to 100% fertilizer levels. The most significant effect was observed at site 2 (1.23%), followed by sites 1 and 3, with a 20% reduced dose of P fertilizer. Compared to the 80% control, P content increased by 9–13% (0.93–1.23%), and compared to the 100% control, it increased by 4–6% (0.97–1.23%). Soil available-P ranged from 5.21–6.72 mg/kg, with the highest contents at site 2 (6.72 mg/kg), followed by site 3 (6.69 mg kg^−1^) and Site 1 (6.19 mg kg^−1^). Overall, soil P availability increased by 9–28% compared to the respective 80% controls (Table 1).

### Detection of inoculated-PSB strains from rice rhizosphere

**Figure 6:**
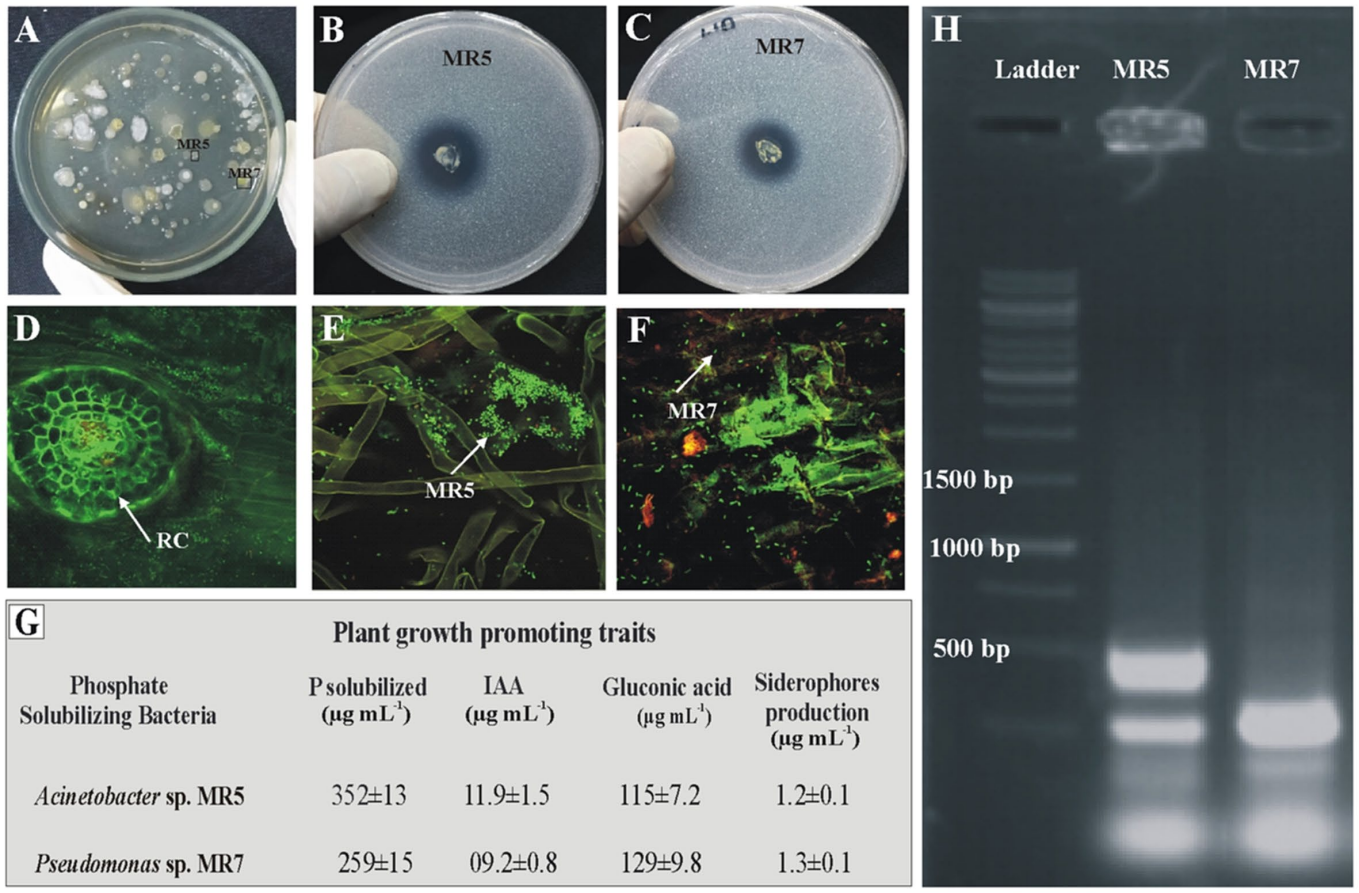
Re-isolation of inoculated PSB colonies **(A)**. Plate showing re-isolated colonies of MR5 and MR7 **(B, C)**. Confocal Laser Scanning Microscopy of rice roots at 35 days post inoculation of P solubilizing bacteria in the microplot experiment under net house conditions. Control treatments without inoculation **(D)** and from the PSB inoculation of MR5 **(E)** and MR7 **(F)**. Plant growth-promoting attributes of re-isolated PSB strains for confirmation of inoculated PSB: P solubilization and IAA production were detected quantitatively by high-performance liquid chromatography (HPLC), gluconic acid production and siderophores production **(G)**. Values are an average of six biological replicates. MR5: *Acinetobacter* sp.’ MR7 and *Pseudomonas* sp. The photograph of the gel indicates re-isolated colonies of PSB that are similar morphologically to the inoculated consortium **(H)**.

Inoculated PSB strains were detected in rice rhizosphere soil from microplot trials. Root colonization by inoculated phosphate solubilizing bacteria was studied using fluorescent *in situ* hybridization through FLUOS-labeled green probe EUB338. BOX-PCR and viable counts were observed 60 days after sowing (Figure 6). BOX fingerprinting amplification confirmed that the identities of the re-isolated PSB colonies matched those of pure PSB cultures. Re-isolated PSB was identified by comparing the morphological characteristics and plant growth-promoting attributes (phosphate solubilization, gluconic acid production, IAA production, and siderophores production) to those of the inoculated bacteria. Re-isolated PSB’s strain-specific fingerprints matched those of pure colonies.

## Discussion

Rice is a highly valued cash crop and holds significant importance as a global staple food. Among rice, the premium quality cylindrical-grained aromatic “Basmati rice” is geographically exclusive to certain districts of India and Pakistan (Dash and Dangar, 2017).

Phosphorus plays an integral role in regulating the metabolism and health of plants but indiscriminate use of phosphate-based chemical fertilizers to meet the plant P requirements in agricultural lands enhanced the residual level of these chemicals in the soil to high solubility and mobility. To the best of our knowledge, a comprehensive study to delineate rice-associated core microbial communities and microbial drivers under diverse field conditions and geographies is completely lacking, as rice microbiome studies are limited to controlled conditions (Wu et al., 2024 Purwestri et al., 2024). To fill this knowledge gap, we explored the microbiome of the field soil to investigate the microbes that already exist or prevail in native soil and then isolate them, optimize the culture conditions for maximum bacterial efficiency in terms of phosphate solubilization, and augment the soil with these bacteria as biofertilizer.

Therefore, we employed a metagenomics technique based on next-generation sequencing to examine the ecology of microorganisms living in their natural habitat, allowing us to connect ecological processes in the environment to specific populations of microorganisms (Urszula, 2022). Understanding the soil microbial community can help in the isolation of soil or site-specific microbes for augmentation in the soil. Unfortunately, the emphasis on deciphering microbial populations from fields and then targeted isolation of required bacterial genera for enhanced nutrient availability of plants is completely missing.

The diversity and distribution of microflora in rice-growing regions differ from those outside this premium zone. This study utilized Illumina sequencing: Performed using the Illumina platform manufactured by (Illumina, Inc., San Diego, CA, USA) of the 16S rRNA gene from rhizospheric soil samples to estimate bacterial diversity at two sites within the rice belt (Sheikhupura and Gujranwala) and one site outside the belt (Faisalabad). Diversity indices, which compare dominance, relative abundance, species richness, and evenness of bacterial species, indicated differences among these sites. Dominant taxonomic groups included Proteobacteria, Acidobacteria, Actinobacteria, Choloflexi, and Firmicutes in all rhizosphere soil samples. Previous studies have also reported the dominance of these groups in the rhizospheric soil of various crops. Proteobacteria were the most abundant, playing a crucial role in nutrient recycling, while Actinobacteria were noted for their importance in nutrient cycling and disease suppression. The study found that bacterial populations in the rice belt were more similar to each other compared to the population at the non-rice belt site.

Previous studies indicated that a 98% relative abundance of Acinetobacter, Enterobacter, and Pseudomonas in leaves and roots of tomato plants (Narsian and Patel, 2009). We detected 15 common genera GpI, *Bradyrhizobium*, *Microvirga*, *Sphingomonas*, *Brevundimonas*, *Pseudomonas*, *Thiobacillus*, *Ramlibacter*, *Syntrophobacter*, *Bacillus*, *Hydrogenispora*, *Sporacetigenium*, *Turicibacter*, *Gp6*, and *Flavobacterium* in both compartments. Similarly, *Massilia, Flavobacterium, Pseudomonas, and Rathayibacter* are found to be a prevalent bacterial genus in the leaves and root zone of *Arabidopsis thaliana* [45]. In the current study, we observed a higher percentage of unclassified sequences in the rhizosphere. Previous results also indicated that the percentage of unknown taxa was higher in the rhizosphere than in the phyllosphere (Amardip Singh et al., 2012). We targeted the isolation of *Pseudomonas*, *Bacillus*, and *Acinetobacter* belonging to biosafety level 1 for soil/seed inoculation for enhanced growth of Basmati rice. Phosphate solubilizing bacteria *Acinetobacter* sp. strain MR5 (GenBank accession number LT629134, and DSMZ accession number DSM 106631) and *Pseudomonas* sp. strain MR7 (GenBank accession number LT629136 and DSMZ accession number DSM 106634) isolated from rice growing area of province Punjab, Pakistan were selected based on their P solubilizing potential.

In soil microcosm with two well-characterized strains *Acinetobacter* sp. MR 5 and *Pseudomonas* sp. MR7 is based on their potential to solubilize insoluble phosphate from soil complexes. Soil microcosm studies authenticate the role of PSB in increasing soil available P and evaluate the survival of PSB in soil. These PSB were found to be effective in enhancing available P concentration in soil, indicating their significant P release up to 60 days period. Maximum phosphatase activity was observed in treatment in which soil was amended with TCP and glucose. The addition of TCP and glucose helped the bacteria to proliferate and survive. As described previously, the addition of an insoluble phosphate source significantly increased the total PSB populations and enhanced the plant P uptake as more soluble P would be released into the soil by PSB for plant uptake (CharanaWalpola and Yoon, 2013). No drastic change was observed in the pH of the soil as observed in *in vitro* broth medium due to the buffering nature of the soil used in the experiment, as Gyaneshwar et al. (2002) reported.

Since an organism’s nutritional and physiological needs are genetically established, it is critical to create an environment that promotes the best possible bacterial activity (Verma et al., 2018). Response Surface Methodology (RSM) provides an alternative methodology to optimize a process by considering the interactions among the factors and giving an estimate of the combined effects of these factors on the final output, which leads to simplification of the optimization process and cheaper cost of production. Using RSM, the critical components’ effects were assessed concurrently for both effective PSB *Acinetobacter* sp. MR5 and *Pseudomonas* sp. MR7.

Results indicate that the tested strains can potentially solubilize phosphorus across a wide range of physical parameters. Researchers have suggested varying optimal temperatures for phosphate solubilization, ranging from 25 to 37°C (Shahab and Ahmed, 2008; Fasim et al., 2002; Kang et al., 2002). Our findings indicate that the optimal solubilization activity is shown by *Acinetobacter* and *Pseudomonas* species at 25°C and an initial pH of 7. Reduced growth occurs below pH 5 and above pH 8, according to FarhatMB and BejorW (2009), indicating that a medium pH of 5–7 is appropriate for solubilization. After optimizing these culture conditions, we used them to re-optimize maximum inoculum production within 36 h. Large-scale inoculum production is essential for field application. The right inoculum size is essential because high density can deplete nutrients or substrate due to excessive biomass synthesis, while low density can result in insufficient biomass and decreased product development (Sharma et al., 2008).

Using TCP as a P source, the *in vitro* biokinetics of two PSB strains, *Acinetobacter* sp. MR5 and *Pseudomonas* sp. MR7 showed that the maximum solubilization happened at 0.038 percent and 0.008% inoculum density, respectively. The *Pseudomonas* sp. MR7 strain grew faster due to its shorter doubling time (*t*_d_), which was 7.8 h. The growth rate of MR5 was lower and it took 19.6 h to double its cells quantity. The rate of P solubilization by MR7 was slowed down by increasing the inoculum density, and at 0.008% (w/v) inoculum level, qp was more than 0.014%. Contrary to this, bacterial strain MR5 showed higher P solubilization at 0.038% inoculum level, the highest among the used inoculum concentrations. The biokinetics study assisted in minimizing the production period of PSB strains, lowering the expense of producing large amounts of inoculum in a short period.

In the current study, evaluation of these strains in microplots showed the effectiveness of optimized inoculum as indicated by a percent increase in plant growth parameters, that is, plant height number of tillers, total biomass, straw yield grain yield, and plant P in plants inoculated with a consortium of these PSB as compared uninoculated controls of 100 and 80% of the recommended dose of chemical P. Suleman et al. (2018) reported the injection of PSB on wheat plant resulted in an increase of 26% in grain yield and 15% in plant P. The optimum soil temperature reported for root development in rice is 25°C (Arai-Sanoh et al., 2010), the same temperature where our bacteria showed maximum P-solubilization activity. This encourages the use of these bacteria as rice inoculum in Pakistani soils. Furthermore, P solubilization activity in diverse conditions (pH and temperature) leads to the conclusion that these bacteria can also be effective for other crops.

The present study has demonstrated that the consortium of PSB is more effective for promoting growth, yield, and plant P uptake than single strain inoculum even with 20% reduced application of P-based chemical fertilizer.

Moreover, evaluation of the PSB consortium across multiple rice-growing sites under various field conditions demonstrated increases in grain yield, straw yield, and plant phosphorus uptake compared to un-inoculated controls. The majority of previous studies evaluated PSB in pot experiments, but few have assessed their impact on plant phosphorus content and yield in field conditions. In this study, selected strains enhanced plant phosphorus content by 13–21% and soil available P by 9–28% in natural environmental conditions. The PCA and CAT-PCA analysis revealed soil-specific responses to bacterial inoculation. This study additionally confirmed the inoculation strains using the gcd gene and other characteristics. Viable cell counts, BOX-PCR, and FISH confirmed the competency and significant effects of the PSB consortium on increasing plant P, soil available P, and grain yield.

## Conclusion

This detailed research study validated the effectiveness of rice rhizosphere-associated phosphate solubilizing bacteria under control and natural soil systems. This study highlights the potential of phosphate-solubilizing bacteria in enhancing soil fertility and promoting Basmati rice growth. Utilizing next-generation sequencing to characterize microbial communities, targeted isolation, and optimization of PSB strains such as *Acinetobacter* sp. MR5 and *Pseudomonas* sp. MR7 demonstrated significant improvements in soil phosphorus availability and plant yield. Field trials confirmed the efficacy of PSB-based biofertilizers, suggesting a sustainable alternative to chemical fertilizers for rice cultivation.

## Data Availability

The datasets presented in this study can be found in online repositories. The names of the repository/repositories and accession number(s) can be found in the article/Supplementary material.

## Footnotes

^1^ https://joey711.github.io/phyloseq/

^2^ https://www.r-project.org/

^3^ https://www.ncbi.nlm.nih.gov/bioproject/574892
