# Supplementary material for: Diversity and functional traits based indigenous rhizosphere associated phosphate solubilizing bacteria for sustainable production of rice
Source: Front Microbiol. 2024 Dec 13;15:1470019. doi: 10.3389/fmicb.2024.1470019 (PMC11671494; doi:10.3389/fmicb.2024.1470019)
Supplement: Supplementary file 1 [file Supplementary_file_1.docx]

**Table S1 Properties of Soil and Water Samples Collected from different areas of Rice Growing Kalar belt of Punjab, Pakistan**

| **Parameter** | **Faisalabad** | **KalaShah Kaku** | **Sheikhupura** |
| --- | --- | --- | --- |
|  |  | **Soil** |  |
| **pH** | 7.5 ±0.25 | 7.5 ±0.5 | 7.70 ±0.35 |
| **Texture** | Loamy sand | Sandy loam | Sandy loam |
| **EC (µs/cm)** | 2900±27.0 | 818 ±19 | 305±24.0 |
| **Total N (%)** | 0.06±0.002 | 0.11±0.001 | 0.10±0.001 |
| **organic matter (%)** | 1.85±0.01 | 3.54±0.02 | 2.81±0.05 |
| **Available NO3 (%)** | 0.01±0.001 | 0.17±0.003 | 0.20±0.002 |
| **Available NH4 (%)** | 0.04±0.000 | 0.06±0.00 | 0.05±0.001 |
| **Total P (ppm)** | 8.83±0.27 | 5.5 ±0.018 | 9.31±1 |
| **Available P (mg Kg^-1^)** | 1.9 ±0.011 | 1.3 ±0.001 | 3.1±0.09 |
| **Zn (ppm)** | 0.15±0.001 | 0.71±0.001 | 0.18±0.001 |
| **Mn (ppm)** | 0.52±0.004 | 0.71±0.003 | 0.61±0.02 |
| **Mg (ppm)** | 0.18±0.006 | 3.8±0.016 | 1.4 ±0.008 |
| **Fe (ppm)** | 1.00±0.002 | 22.4±1.87 | 8.6 ±0.7 |
|  |  | **water** |  |
| **pH** | 7.79±0.5 | 7.6 ±0.28 | 7.69 ±0.43 |
| **EC (µs/cm)** | 1096±15 | 1003±7 | 980 ±16 |
| **TDS (mg L^-1^)** | 701 ±2.3 | 641±8.9 | 627 ±10.02 |

#### Table S2: Effect of Inoculum Density on Kinetics of *In Vitro* Phosphate Solubilization By

***Acinetobacter* sp. MR5 and *Pseudomonas* sp. MR7 in Liquid Broth**

| **Phosphate solubilizing bacteria** | **Inoculum density**  **% (w/v)** | **P Solubilization (mg dL^-1^)** | **Cell Mass**  **(g dL^-1^)** | ***µ***  **(h^-1^)** | **td**  **(h)** | ***Y*p/x**  **(mg g-1)** | ***q*p**  **(mg g^-^**  **1 h-1)** | **R**  **Value** |
| --- | --- | --- | --- | --- | --- | --- | --- | --- |
| *Acinetobacter* | 0.022 | 8.68 ± 0.43 | 0.27±  0.013 | 0.018 | 38.3 | 32.13 | 0.58 | 0.998 |
| sp. MR5 | 0.038 | 10.86± 0.54 | 0.34±  0.017 | 0.035 | 19.6 | 31.78 | 1.12 | 0.998 |
| *Pseudomonas* | 0.008 | 9.36± 0.46 | 0.32±  0.015 | 0.035 | 19.7 | 29.47 | 1.04 | 0.997 |
| sp. MR7 | 0.014 | 7.82± 0.39 | 0.72±  0.035 | 0.088 | 7.8 | 10.86 | 0.96 | 0.997 |

*Acinetobacter* sp. MR5 (0.022% and 0.038% inoculum w/v wet cells) and *Pseudomonas* sp. MR7 (0.008% and 0.014% inoculum w/v wet cells) were used for inoculation in Pikovskaya broth and kept at 28 ± 2 ᵒC. Supernatant was collected for different activity. Means are an average of three biological replicates, ± represents standard error. td: doubling time of cell biomass, *µ*: specific growth rate, *Yp*/x: product (cell mass) yield coefficient, *q*p: specific rate of product formation

Table S3. Evenness, Diversity and Richness of Rhizosphere soil Sample of Basmati Rice at 3% Sequence Divergence.

| **Sites** | **N** | **Clusters** | **Chao** | **H'** | **varH** | **E** |
| --- | --- | --- | --- | --- | --- | --- |
| **Sheikhupura** | 8410 ± 485A | 2338 ± 226 A | 2339 ±226 A | 7.01 ± 0.17 A | 0.0003 A | 0.90 ± 0.017 A |
| **Gujranwala** | 5174 ± 2306 B | 1488 ± 744 B | 1489 ±743 B | 6.59 ±0.41 A | 0.0005 A | 0.91 ± 0.015 A |
| **Faisalabad** | 6485 ± 1128 AB | 1327 ±509 B | 1330 ± 507 B | 6.27 ±0.44 A | 0.0004 A | 0.88 ±0.007 B |

± showed standard deviation of three replicates. Alpha diversity within each community using two commonly used metrics. Richness (Chao) measures how many unique OTUs were detected in each sample while Shannon-entropy measures randomness or uncertainty in the community. E, Shannon Wiener equitability index; H´, Shannon-Wiener index; varH', the variance of H. Gujranwala (site 3) and Sheikhupura (site 1) were two Basmati rice-growing areas while Faisalabad (site 2) was selected for a comparative study of the diversity of bacterial

population with that of non-Basmati growing area. Means followed by the same letter differ non-significantly at p = 0.05 according to LSD. Different letters show statistical significance of treatments while similar letters show non-significant differences.

**Table S4** CCD matrix showing actual and predicted value of phosphate solubilization using *Acinetobacter* sp. MR5

| Standard no. | Variables in un-coded level | | | Response  (phosphate solubilization) | |
| --- | --- | --- | --- | --- | --- |
|  | A | B | C | Actual | Predicted |
| 1 | 7 | 37.5 | 0.6 | 100 | 96.82 |
| 2 | 7 | 37.5 | 0.6 | 92 | 96.82 |
| 3 | 7 | 37.5 | 0.6 | 89 | 96.82 |
| 4 | 5.3 | 37.5 | 0.6 | 61 | 62.91 |
| 5 | 8 | 45.0 | 0.7 | 69 | 68.00 |
| 6 | 7 | 24.8 | 0.6 | 160 | 167.82 |
| 7 | 8 | 30 | 0.5 | 120 | 122.01 |
| 8 | 8.6 | 37.5 | 0.6 | 75 | 76.71 |
| 9 | 8 | 30.0 | 0.7 | 70 | 65.85 |
| 10 | 7 | 37.5 | 0.43 | 65 | 66.76 |
| 11 | 7 | 37.5 | 0.6 | 100 | 96.82 |
| 12 | 7 | 50.1 | 0.6 | 99 | 100.80 |
| 13 | 7 | 37.5 | 0.6 | 102 | 96.82 |
| 14 | 6 | 30.0 | 0.7 | 110 | 107.64 |
| 15 | 8 | 45.0 | 0.5 | 88 | 96.16 |
| 16 | 6 | 45.0 | 0.7 | 49 | 53.79 |
| 17 | 6 | 45.0 | 0.5 | 35 | 37.96 |
| 18 | 7 | 37.5 | 0.76 | 30 | 32.85 |
| 19 | 7 | 37.5 | 0.6 | 102 | 96.82 |
| 20 | 6 | 30 | 0.5 | 118 | 119.80 |

**Table S5** CCD matrix showing actual and predicted value of phosphate solubilization using *Pseudomonas* sp. MR7

| Standard no. | variables | | | Response  Phosphate solubilization | |
| --- | --- | --- | --- | --- | --- |
|  | **A** | **B** | **C** | **Actual** | **Predicted** |
| 1 | 8.00 | 30.00 | 0.50 | 93 | 91.33 |
| 2 | 7.00 | 37.50 | 0.43 | 69 | 67.54 |
| 3 | 6.00 | 45.00 | 0.70 | 86 | 90.86 |
| 4 | 6.00 | 30.00 | 0.50 | 62 | 64.50 |
| 5 | 6.00 | 45.00 | 0.50 | 63 | 69.62 |
| 6 | 7.00 | 37.50 | 0.60 | 120 | 119.29 |
| 7 | 7.00 | 37.50 | 0.60 | 118 | 119.29 |
| 8 | 5.32 | 37.50 | 0.60 | 59 | 58.15 |
| 9 | 6.00 | 30.00 | 0.70 | 87 | 89.25 |
| 10 | 7.00 | 50.11 | 0.60 | 62 | 63.09 |
| 11 | 7.00 | 37.50 | 0.60 | 118 | 119.29 |
| 12 | 7.00 | 37.50 | 0.60 | 121 | 119.29 |
| 13 | 8.00 | 30.00 | 0.70 | 115 | 116.57 |
| 14 | 7.00 | 37.50 | 0.77 | 104 | 106.64 |
| 15 | 7.00 | 37.50 | 0.60 | 119 | 119.29 |
| 16 | 8.00 | 45.00 | 0.70 | 44 | 43.68 |
| 17 | 8.68 | 37.50 | 0.60 | 39 | 41.03 |
| 18 | 8.00 | 45.00 | 0.50 | 26 | 21.94 |
| 19 | 7.00 | 37.50 | 0.60 | 116 | 119.29 |
| 20 | 7.00 | 24.89 | 0.60 | 118 | 120.09 |
