# Supplementary material for: Diversity and functional traits based indigenous rhizosphere associated phosphate solubilizing bacteria for sustainable production of rice
Source: Front Microbiol. 2024 Dec 13;15:1470019. doi: 10.3389/fmicb.2024.1470019 (PMC11671494; doi:10.3389/fmicb.2024.1470019)
Supplement: Supplementary file 2 [file Supplementary_file_2.docx]

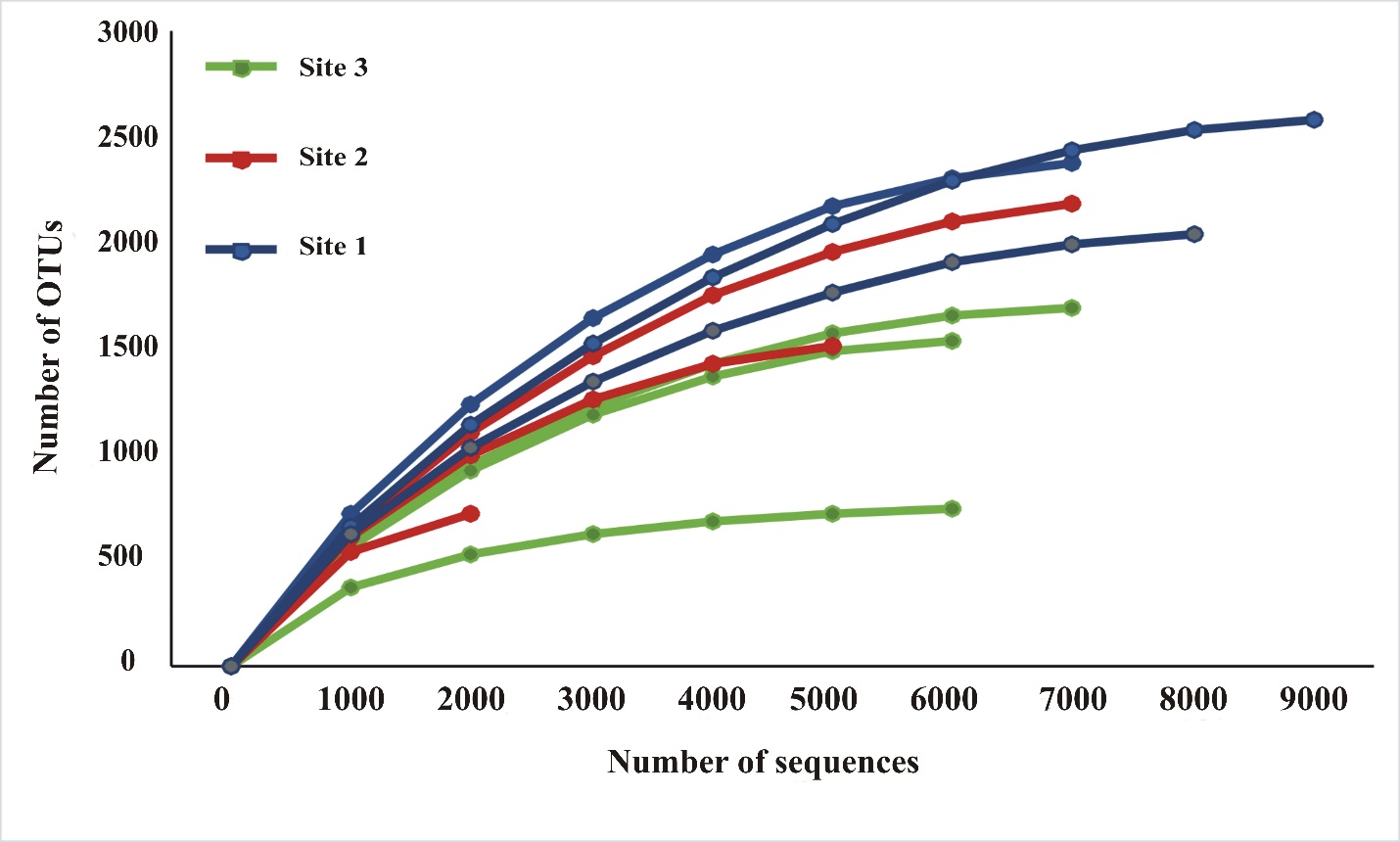


**Figure S1: Rarefaction Analysis of Rhizospheric Soil Sample of Basmati Rice collected from Basmati Rice Growing Areas of Kalar Belt.**


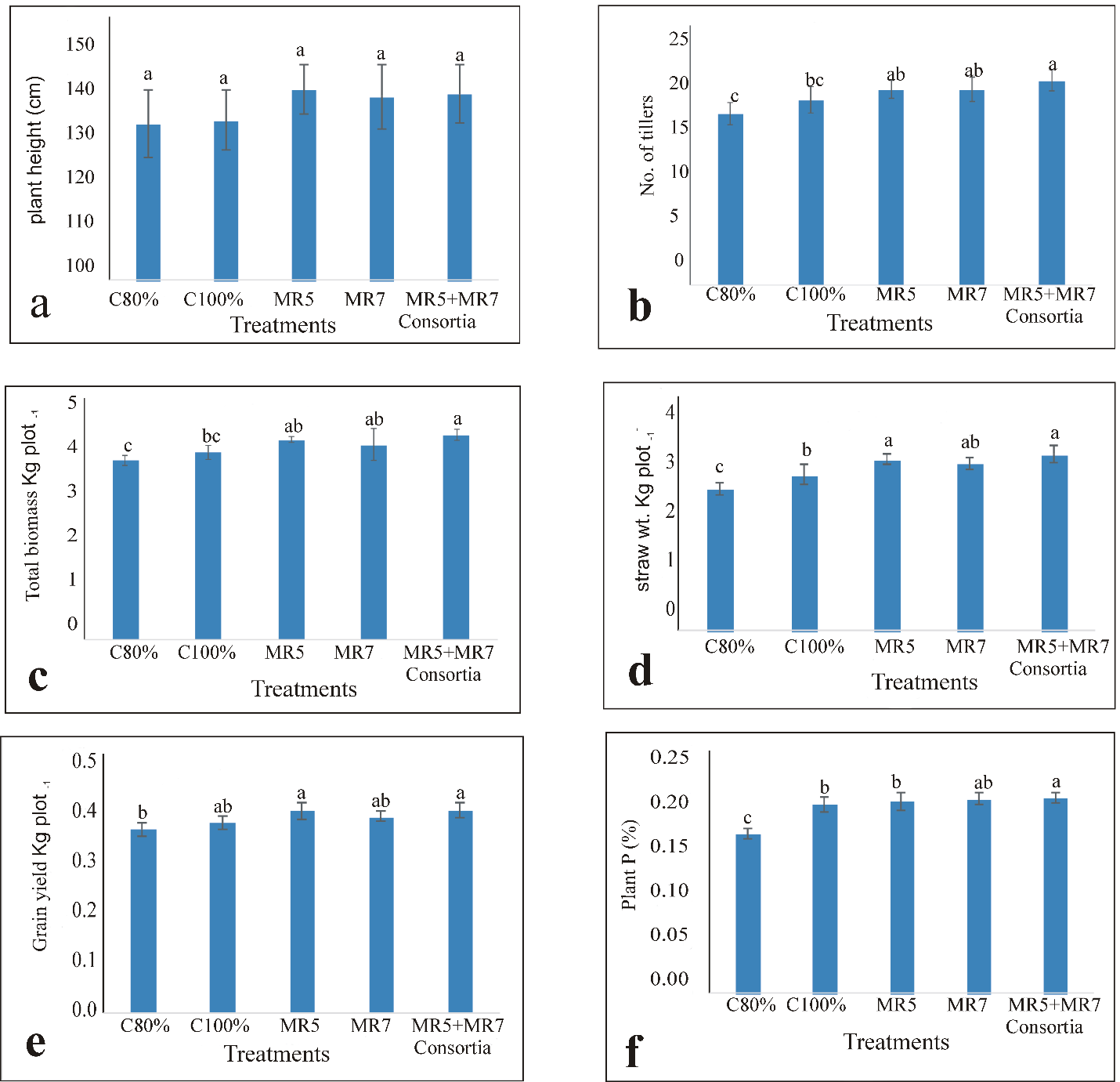


**Figure S2**: ***In Vivo* Evaluation of Phosphate Solubilizing Bacteria for Plant Growth Promotion and Plant P Uptake.** (A) Plant height, (B) Number of Tillers, (C) Total Plant Biomass (Kg Plot -1 ), (D) Straw Weight (Kg Plot -1 ), (E) Grain Yield (Kg Plot -1 ) and (F) Plant P Contents (%) of rice grown in microplots under net house conditions.
